# Serum Amyloid A Production Is Triggered by Sleep Deprivation in Mice and Humans: Is That the Link between Sleep Loss and Associated Comorbidities?

**DOI:** 10.3390/nu9030311

**Published:** 2017-03-21

**Authors:** Edson M. de Oliveira, Bruna Visniauskas, Sergio Tufik, Monica L. Andersen, Jair R. Chagas, Ana Campa

**Affiliations:** 1 Departamento de Análises Clínicas e Toxicológicas, Universidade de São Paulo, Av. Prof. Lineu Prestes, 580, São Paulo SP 05509-000, Brazil; edson.fbq@gmail.com; 2 Departamento de Psicobiologia, Universidade Federal de São Paulo, Rua Napoleão de Barros, 925, São Paulo SP 04024-002, Brazil; brunavisniauskas@gmail.com (B.V.); sergio.tufik@unifesp.br (S.T.); ml.andersen12@gmail.com (M.L.A.); jchagas1@gmail.com (J.R.C.)

**Keywords:** sleep curtailment, sleep loss, obesity, type 2 diabetes, SAA

## Abstract

Serum amyloid A (SAA) was recently associated with metabolic endotoxemia, obesity and insulin resistance. Concurrently, insufficient sleep adversely affects metabolic health and is an independent predisposing factor for obesity and insulin resistance. In this study we investigated whether sleep loss modulates SAA production. The serum SAA concentration increased in C57BL/6 mice subjected to sleep restriction (SR) for 15 days or to paradoxical sleep deprivation (PSD) for 72 h. Sleep restriction also induced the upregulation of *Saa1.1*/*Saa2.1* mRNA levels in the liver and *Saa3* mRNA levels in adipose tissue. SAA levels returned to the basal range after 24 h in paradoxical sleep rebound (PSR). Metabolic endotoxemia was also a finding in SR. Increased plasma levels of SAA were also observed in healthy human volunteers subjected to two nights of total sleep deprivation (Total SD), returning to basal levels after one night of recovery. The observed increase in SAA levels may be part of the initial biochemical alterations caused by sleep deprivation, with potential to drive deleterious conditions such as metabolic endotoxemia and weight gain.

## 1. Introduction

Obesity is now reaching pandemic proportions across much of the world and its consequences includes unprecedented health, social and economic issues. Several aggravating factors have been identified and are considered contributors to the current epidemic of obesity. Sleep disorders are among the factors that raised more concerns (for review see [1,2]).

Sleep loss induces metabolic and endocrine alterations, such as decreased glucose tolerance, decreased insulin sensitivity, increased concentrations of cortisol and ghrelin, decreased levels of leptin, and increased hunger and appetite [2]. Although these metabolic and endocrine alterations are frequently used to support a causal relationship between sleep loss and obesity/insulin resistance, it is still missing the identification of a triggering factor for weight gain in sleep disorders.

The difficulty of translating findings directly from animal models to humans and the challenge of finding an experimental model that leads to weight gain are some of the elements that prevent new discoveries regarding the mechanisms involved in weight gain led by sleep loss. Recently, using the multiple platform sleep restriction (SR) experimental model, we were able to link a past history of sleep restriction to subsequent complications arising from a high-fat diet [3].

Several possible causes linking reduced sleep and obesity, such as neuroendocrine changes, increased food intake, decreased energy expenditure and circadian disruption, share an inflammatory status as a common factor. Here, we focused in a specific inflammatory protein that has progressively gained recognition for its role in the obesity process, the acute phase protein serum amyloid A (SAA). Recently, we used SAA-targeted antisense oligonucleotide (ASO_SAA_) on a high-fat diet-induced obesity model and identified SAA as an additional trigger driving endotoxemia, weight gain and insulin signaling impairment [4].

SAA production is upregulated in the liver and adipose tissue in the acute inflammatory process and it has been considered to have a role in the activation of immune cells triggering inflammatory responses [5]. Moreover, SAA has growth factor–like activity, such as increasing the proliferation of different cell types, including preadipocytes [6,7]. SAA is also able to bind to members of the Toll-like receptors (TLRs) family that are involved in the inflammatory process and metabolic control in obesity [8,9]. Here, we addressed if the production of SAA is one of the biochemical factors present in sleep restriction.

## 2. Materials and Methods

Animals. Male C57BL/6 mouse (three months of age) from CEDEME Universidade Federal de São Paulo (UNIFESP), housed in a room maintained at 20 ± 2 °C in 12:12 h light/dark cycle, chow diet (Nuvilab CR-1, Colombo, Brazil) and water *ad libitum*, were submitted to sleep restriction (SR) or paradoxical sleep deprivation (PSD) protocols. For each experimental group, six to 12 animals were used. The experimental protocol was approved by the Ethical Committee of UNIFESP (approval No. 0474/09). The euthanasia occurred immediately after each last experimental period by anesthesia overdose (i.p. administration of a combination of ketamine (100 mg/kg) and xylazine (15 mg/kg)), and ensured by cervical dislocation. During terminal anesthesia, serum samples were collected by cardiac puncture.

Sleep restriction (SR) protocol. The SR method used in this study was an adaptation of the multiple platform method, originally developed for rats [10], and performed as previously described [3,11,12]. The animals were randomly assigned into control group and SR group. The SR group was sleep restricted for 15 days, 21 h daily. After each 21 h period of SR, the mice were allowed to sleep for 3 h (sleep opportunity beginning at 10 a.m.).

Paradoxical sleep deprivation (PSD) and paradoxical sleep rebound (PSR) protocols. For PSD experiments, the animals were randomly assigned into three groups: the control group, the PSD group and the PSR group. PSD animals were sleep deprived for 72 consecutive hours, using the multiple platform method as previously described [3,13,14,15]. The PSR mice were sleep deprived for 72 consecutive hours followed by 24 h in sleep rebound period. A home cage control group was in the same room for the duration of the experiment, sleeping ad libitum. During the 72 h PSD period mice were placed inside a water tank (41 cm × 34 cm × 17.5 cm), filled up to 1 cm of the upper border and containing 12 circular platforms, 3.5 cm in diameter. Animals could thus move around inside the tank by jumping from one platform to another. When PSD was reached, muscle atonia set in, animals fell into the water and woke up. Food and water were provided ad libitum. Water in the tank was changed daily throughout the study period.

Human sleep deprivation. The experimental protocol was performed as previously described [16,17] with 30 healthy male volunteers ranging from 19 to 29 years. The exclusion criteria included sleep disorders, shift work, extreme morningness-eveningness, neurological or psychiatric diseases, smoking and alcohol or substance abuse, including any medicine able to change sleep patterns. The participants had normal results on Pittsburgh Sleep Quality Index, Epworth Sleepness Scale and Beck Depression Inventory. Briefly, three experimental groups were randomly assigned (10 non-sleep deprived, 10 total sleep deprived, and 10 REM sleep deprived) and the protocol was performed on nine consecutive days: one adaptation night, one baseline night, two nights of total sleep deprivation (Total SD) or four nights of REM sleep deprivation (REM SD), followed by three nights of sleep recovery for both groups. The control group was also maintained in the laboratory during the entire experimental protocol and had regular nights of sleep monitored by polysomnography (uninterrupted sleep showing normal sleep patterns). All subjects remained in the research center throughout the study period respecting a bedtime schedule (from 11 p.m. to 8 a.m.), in accordance with their regular habits (7–9 h sleep per night), and receiving 4 meals per day [17]. Total SD volunteers could read, play games, watch television or ambulate within the building to help them stay awake. Therefore, we cannot exclude circadian interference on SAA levels. The subjects were also abstained from running or any other type of resistance exercise. The investigators were continuously present to monitor wakefulness to ensure that subjects would not fall asleep during the study. REM SD volunteers were awakened when observed desynchronized EEG without spindles or K complexes and the concomitant reduction of the tonic electromyogram amplitude. The volunteers were kept awake for a sufficient time to avoid an immediate relapse into REM sleep while keeping the waking episodes short enough to allow frequent interventions. The study was conducted at the Sleep Laboratory of the Department of Psychobiology at the Universidade Federal de São Paulo (UNIFESP) with the approval of the Ethics Committee of the University (#1163/2016). All participants provided informed consent prior to enrolling in the study. Blood samples were obtained every morning (8 a.m.) during the experimental protocol (baseline, two nights of Total SD, four nights of REM SD and three nights of recovery). Blood samples were centrifuged immediately at 4 °C, and then the plasma were stored at −80 °C until the assays were conducted.

Quantitative real-time PCR. Total RNA from epididymal adipose tissue and liver was isolated using Qiagen RNeasy^®^ Lipid Tissue Mini kit (Qiagen, Hilden, Germany). cDNA was then synthesized from 1 μg of RNA using the High Capacity cDNA Reverse Transcription (Life Technologies^®^, Grand Island, NY, USA). Real-time PCR were performed using SyBr^®^ Green Master Mix (Life Technologies^®^, Grand Island, NY, USA) for *Saa1.1/2.1* (F-5′-AGA CAA ATA CTT CCA TGC TCG G-3′ and R-5′-CAT CAC TGA TTT TCT CAG CAG C-3′). Real-time PCR for *Saa3* was performed using the TaqMan^®^ assay (Applied Biosystems^®^, Grand Island, NJ, USA), catalogue number Mm00441203_m1–*Saa3* and β-actin (*Actb*), number 4552933E, as an endogenous housekeeping gene control. Relative gene expression was determined using the 2^−ΔΔCt^ method [18].

SAA and endotoxin quantification. Serum/plasma concentrations of SAA were determined using ELISA following the manufacturer’s instructions: mouse SAA (Tridelta Development Ltd., Maynooth, Ireland) and human SAA (Invitrogen^®^, Camarillo, CA, USA). Endotoxin was measured with the limulus amoebocyte lysate (LAL) chromogenic endpoint assay (Lonza, Allendale, NJ, USA).

Immunofluorescence. Using paraffin-embedded sections (5 μm thick) from epididymal adipose tissue, immunofluorescence for SAA was performed using a rabbit anti-mouse SAA (1:200 dilution, kindly produced and provided by De Beer laboratory, University of Kentucky, KY, USA) [19], subsequently incubated with the appropriate secondary fluorescent antibody (Invitrogen^®^, Camarillo, CA, USA) and the slides mounted using Vectashield set mounting medium with 4,6-diamidino-2-phenylindol-2-HCl (DAPI; Vector Laboratories Inc., Burlingame, CA, USA). An isotype control was used to ensure antibody specificity in each staining. Tissue sections were observed with a Nikon Eclipse 80i microscope (Nikon^®^, Tokyo, Japan) and digital images were captured with NIS-Element AR software (Nikon^®^, Tokyo, Japan).

**Statistical analysis**. Results were presented as mean ± SEM and the number of independent experiments is indicated in each graph. Statistical analysis was performed with Graph Pad Prism4 (Graph Pad Software, Inc., San Diego, CA, USA). When multiple samples were compared with one independent variable, one-way analysis of variance with Newman-Keuls post hoc test was performed. The level of significance was set at *p* < 0.05.

## 3. Results

**Figure 1:**
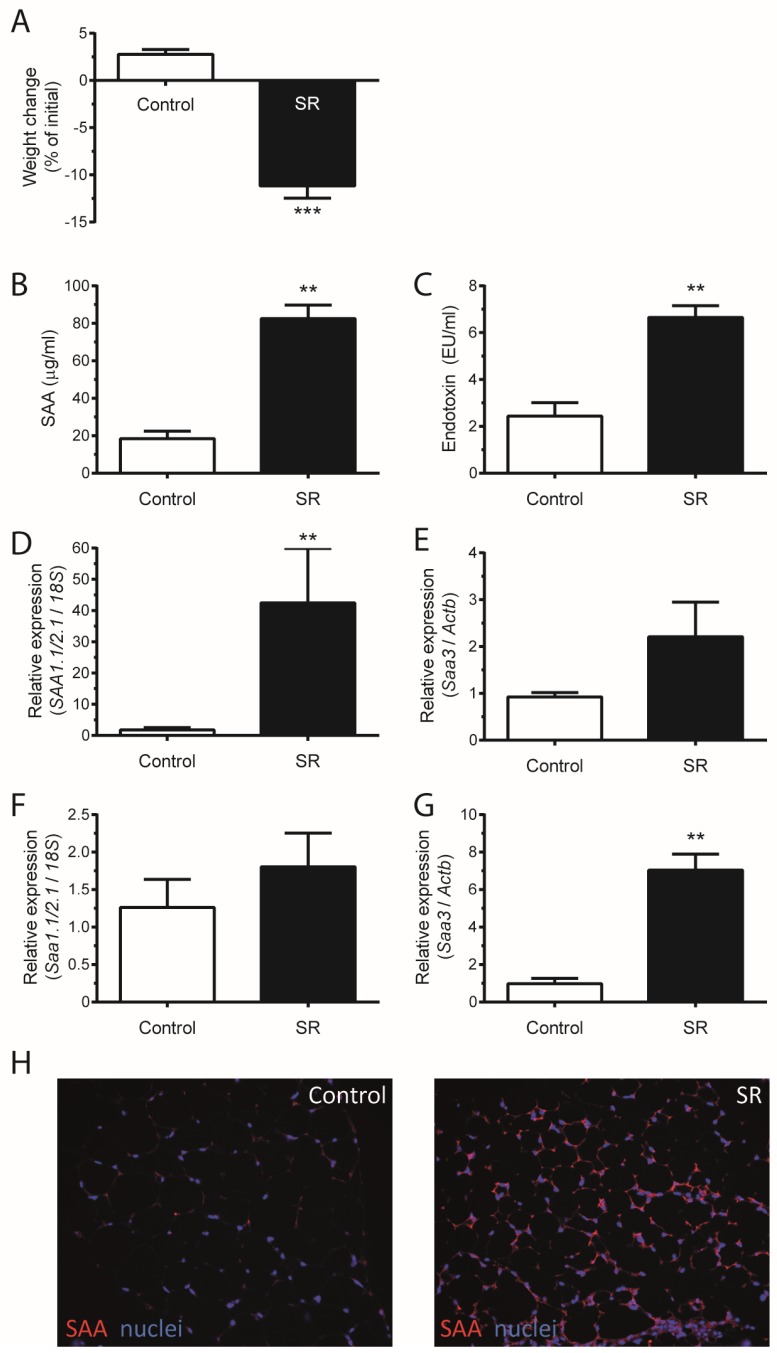
Sleep restriction (SR) causes weight loss and increased SAA production. Mice C57BL/6 were submitted to SR for 21 h daily for 15 days. (**A**) Mice weight change after SR; (**B**) SAA and (**C**) endotoxin concentration in mice serum; Real-time PCR was performed to assess mRNA expression of (**D**) *Saa1.1/2.1* and (**E**) *Saa3* in liver and (**F**) *Saa1.1/2.1* and (**G**) *Saa3* in adipose tissue; (**H**) Control and SR mice adipose tissue stained for SAA. Data are means ± SEM from six to 12 mice per group, with statistical analyses performed by one-way ANOVA followed by Newman-Keuls post hoc test (** *p* < 0.01, *** *p* < 0.001, vs. control).

Mice subjected to sleep restriction (SR) for 21 h daily during 15 days lost weight (Figure 1A). After seven and 15 days of SR, mice lost approximately 7% and 12% of weight, respectively. SR led to an increase in serum SAA and endotoxin of approximately four times (Figure 1B,C). *Saa1.1/2.1* and *Saa3* are inducible tissue-specific isoforms in the liver and adipose tissue, respectively [5,20,21]. Sleep restriction led to the mRNA expression of tissue-specific isoforms of SAA (Figure 1D–G). In the liver, the isoform *Saa1.1/2.1* was upregulated 10–40 times (Figure 1D) and *Saa3* remained unaltered (Figure 1E). In adipose tissue, whereas no difference in *Saa1.1/2.1* expression was observed (Figure 1F), the isoform *Saa3* was upregulated around seven times (Figure 1G). The data was confirmed by immunostaining the adipose tissue, where SAA production was induced after SR (Figure 1H).

**Figure 2:**
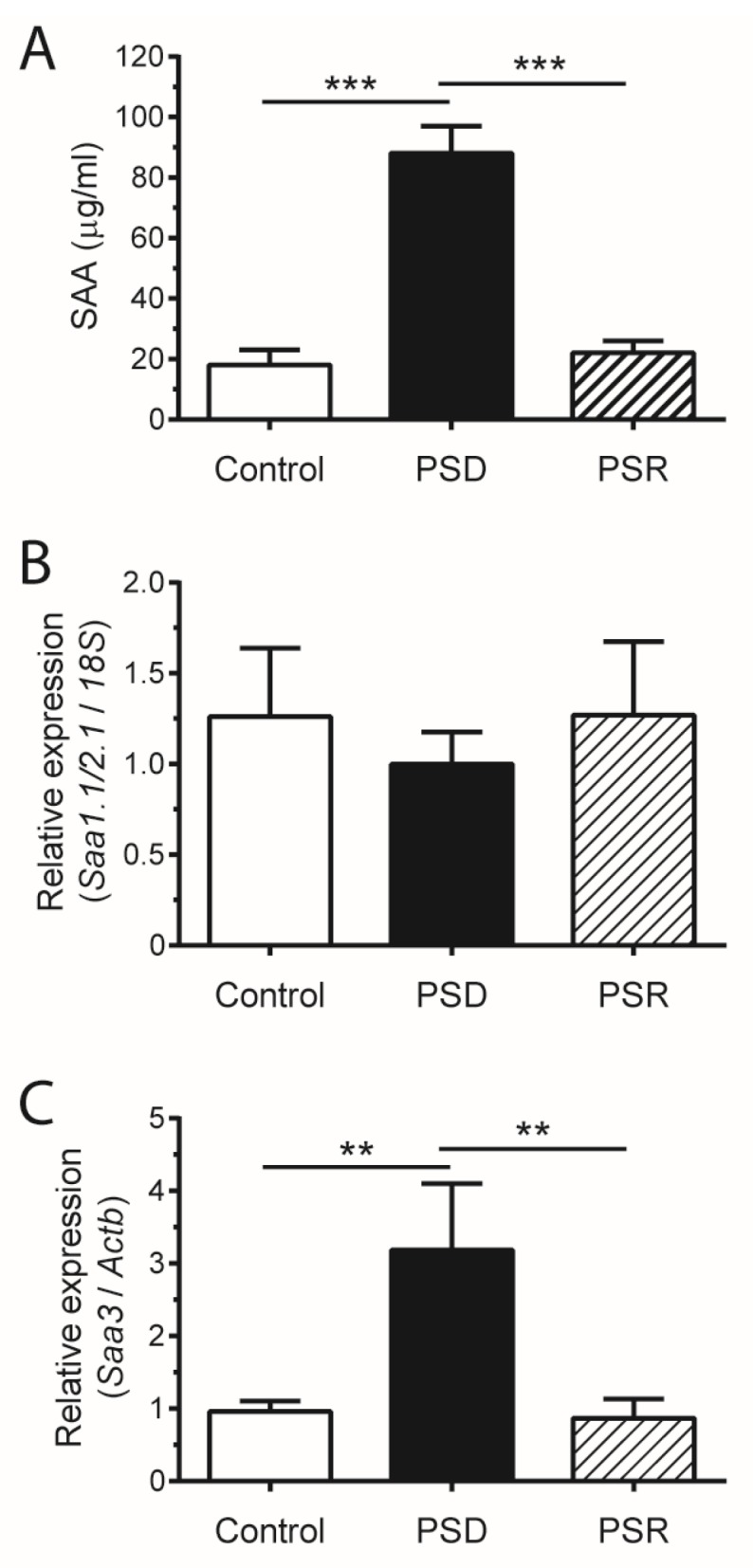
Paradoxical sleep deprivation (PSD) increases SAA levels in a stimulus-dependent manner. C57BL/6 mice were submitted to PSD for 72 uninterrupted hours, followed by a 24 h paradoxical sleep rebound (PSR) period. (**A**) SAA concentration in serum. Real-time PCR was performed to assess mRNA expression of (**B**) *Saa1.1/2.1* and (**C**) *Saa3* in adipose tissue. Data are means ± SEM from six mice per group, with statistical analyses performed by one-way ANOVA followed by Newman-Keuls post hoc test (** *p* < 0.01, *** *p* < 0.001, vs. control).

Paradoxical sleep deprivation (PSD) and paradoxical sleep rebound (PSR) were primarily used to assess the extent, severity and length of the SAA increment induced by sleep loss. Similarly to sleep restriction (SR), mice subjected to paradoxical sleep deprivation (PSD) also showed an increase in serum SAA levels (four times higher than control mice) (Figure 2A). Besides that, PSD mice showed no difference in *Saa1.1/2.1* expression (Figure 2B) in the adipose tissue, with *Saa3* mRNA being upregulated about three times (Figure 2C). Interestingly, PSD regulates SAA production in a stimulus-dependent manner, once it was observed that SAA serum levels and *Saa3* mRNA expression in adipose tissue returned to baseline after the rebound period (Figure 2A,C).

**Figure 3:**
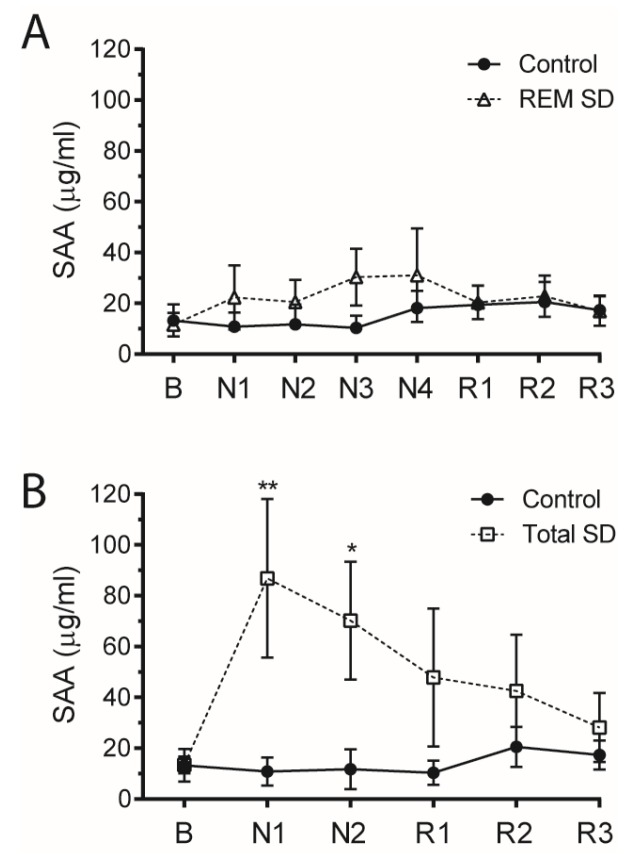
Total sleep deprivation increases plasma SAA in human. Thirty healthy male volunteers aged between 19 to 29 years were randomly assigned to one of three experimental groups after providing a written informed consent (10 in a non-sleep-deprived group (Control), 10 in a total sleep-deprived group (Total SD), and 10 in an REM-sleep-deprived group (REM SD)). Exclusion criteria included the following: sleep disorders, obesity and obstructive sleep apnea (OSA). Plasma SAA concentration in (**A**) REM SD and (**B**) Total SD. Data are means ± SEM (* *p* < 0.05, ** *p* < 0.01).

Finally, it was also possible to measure SAA from plasma derived from healthy human volunteers subjected to two nights of total sleep deprivation (Total SD) or four days in REM sleep deprivation (REM SD) (Figure 3). Although there was no difference regarding the SAA concentration when comparing the control and REM SD groups (Figure 3A), a remarkable four-fold increase in plasma SAA levels was observed after 24 or 48 h of total sleep deprivation (Total SD) (Figure 3B). Interestingly, the SAA levels also returned to basal levels after one night of recovery, showing that Total SD regulates SAA production in humans (Figure 3B).

## 4. Discussion

The purpose of this study was to determine whether sleep restriction is associated with SAA production. We found that sleep restriction led to an increase in the production of SAA in both mice and humans.

In obesity and diabetes, the increment in SAA serum levels reaches no more than a three-fold increase from baseline [5,22]. Considering that, the four-fold elevation in serum SAA observed in sleep restriction (21 h daily for 15 days) and also in paradoxical sleep deprivation (72 consecutive hours) seems a striking result. Moreover, sleep restriction also caused an increment in lipopolysaccharide (LPS) in serum, achieving levels similar to that observed in metabolic endotoxemia, defined as a mild increment of LPS in serum after a short time on a high-fat diet, associated with the onset of diabetes and obesity [23].

Although it was not possible to identify which specific SAA isoform was increased in the mice serum, it is expected to be the hepatic-induced isoforms *Saa1.1/2.1*, once *Saa3* does not contribute to circulating SAA levels [5,21,24]. SAA3 is related to adipose tissue inflammation and its expression regulation may involve a direct induction by the hepatic isoforms *Saa1.1/2.1* [6,25].

The interplay between SAA and LPS has been suggested to play a role in the adipose tissue, making it prone to hypertrophy and consequent weight gain [4]. Both SAA and LPS are able to cause morphologic changes in the adipose tissue, such as promoting preadipocyte proliferation and tissue inflammation [6,26,27]. Despite the direct induction of migration, adhesion and tissue infiltration of monocytes and polymorphonuclear leukocytes [28], SAA induces the release of other chemoattractive cytokines such as MCP-1 and CCL20 [26,29]. Although sleep restriction led momentarily to weight loss, the increase in *Saa3* expression in adipose tissue may be an important factor to trigger obesity and insulin resistance. Our previous study in mice showed that sleep restriction predisposed them to weight gain and insulin resistance, aggravating the harmful effects of a high-fat diet [3].

Sleep restriction and sleep reestablishment seemed to be a prompt regulator for *Saa3* expression in mice adipose tissue. In a stimulus-dependent manner, an increase in *Saa3* expression was observed in PSD, with a rapid return to the baseline expression after 24 h in recovery.

The increment in SAA observed in humans submitted to two nights of total sleep deprivation was similar to that found in mice submitted to sleep restriction. This increment may be due to an increased SAA production or even due to a reduction in SAA clearance. Besides physiological differences between mice and humans, especially related to sleep habits, the increase in serum levels of SAA in response to sleep restriction seems to be similar between the species. However, if paradoxical sleep deprivation in mice was also able to modulate SAA production, no effect was observed when REM sleep deprivation was applied to humans. It is important to highlight that SAA was already described as being altered in obese patients with obstructive sleep apnea syndrome (OSAS) [30], possibly as a consequence of hypoxia/reoxygenation related to sleep apnea [31]. Our findings point out that even in lean and non-OSAS humans, SR is able to increase SAA levels and it may be related to the onset of subclinical inflammation, weight gain and insulin resistance. Moreover, the elevation in circulating non-esterified fatty acid (NEFA) is another event derived from SR [32] and it might be a direct effect of serum amyloid A, once it is able to induce lipolysis [6,26]. Both SAA and NEFA can lead to insulin resistance and play a central role in the development of metabolic diseases [4,32].

In addition to the role of SAA in obesity and insulin resistance, elevated serum levels of SAA is an independent and strong predictor of coronary artery disease and adverse cardiovascular outcome [33]. More recently, clearer evidence of the involvement of SAA in cardiovascular disease (CVD) showed that a brief elevation in SAA levels is sufficient to increase atherosclerosis [34]. Although no measurement of a direct cardiovascular disease risk factor was taken, it should be addressed in future studies.

## 5. Conclusions

In summary, our data show that sleep deprivation triggers SAA production in healthy and non-obese mice and humans. Interestingly, the transient increment in SAA levels occurred simultaneously to a metabolic endotoxemia. These results support the continued investigation of the role of SAA in metabolic diseases and also suggest that increased levels of SAA may be part of the signaling linking sleep loss to its associated comorbidities, such as obesity and type 2 diabetes.
